# Treatment effects of the traditional Chinese medicine Shenks in bleomycin-induced lung fibrosis through regulation of TGF-beta/Smad3 signaling and oxidative stress

**DOI:** 10.1038/s41598-017-02293-z

**Published:** 2017-05-22

**Authors:** Haiyan Chu, Ying Shi, Shuai Jiang, Qicheng Zhong, Yongqiang Zhao, Qingmei Liu, Yanyun Ma, Xiangguang Shi, Weifeng Ding, Xiaodong Zhou, Jimin Cui, Li Jin, Gang Guo, Jiucun Wang

**Affiliations:** 1 State Key Laboratory of Genetic Engineering, Collaborative Innovation Center for Genetics and Development, School of Life Sciences, Fudan University, 2005 Songhu Road, Shanghai, 200438 P. R. China; 2 Department of Rheumatology and Immunology, Yiling Affiliated Hospital of Hebei Medical University, Shijiazhuang, 050091 China; 3 Department of Traditional Chinese Medicine, Geriatric Hospital of Hebei Province, Shijiazhuang, 050011 China; 4 University of Texas Health Science Center at Houston, 6431 Fannin St., Houston, Texas 77030 USA; 5 Institute of Rheumatology, Immunology and Allergy, Fudan University, Shanghai, 200040 P. R. China

## Abstract

Pulmonary fibrosis is a kind of devastating interstitial lung disease due to the limited therapeutic strategies. Traditional Chinese medicine (TCM) practices have put forth Shenks as a promising treatment approach. Here, we performed *in vivo* study and *in vitro* study to delineate the anti-fibrotic mechanisms behind Shenks treatment for pulmonary fibrosis. We found that regardless of the prophylactic or therapeutic treatment, Shenks was able to attenuate BLM-induced-fibrosis in mice, down regulate extracellular matrix genes expression, and reduce collagen production. The aberrantly high Smad3 phosphorylation levels and SBE activity in TGF-β-induced fibroblasts were dramatically decreased as a result of Shenks treatment. At the same time, Shenks was able to increase the expression of antioxidant-related genes, including *Gclc* and *Ec-sod*, while reduce the transcription levels of oxidative-related genes, such as *Rac1* and *Nox4* demonstrated by both *in vivo* and *in vitro* studies. Further investigations found that Shenks could decrease the oxidative productions of protein (3-nitrotyrosine) and lipid (malondialdehyde) and increase GSH content both in bleomycin treated mouse lungs and TGF-β stimulated fibroblasts, as well as inhibit the production of ROS stimulated by TGF-β to fight against oxidative stress. Overall, Shenks inhibited fibrosis by blocking TGF-β pathway and modulating the oxidant/antioxidant balance.

## Introduction

Pulmonary fibrosis, including idiopathic pulmonary fibrosis (IPF) and interstitial lung fibrosis (secondary to conditions such as SSc and rheumatoid arthritis) are particularly severe lung diseases characterized by epithelial injury, impaired wound healing and accumulation of fibroblasts as well as extracellular matrix (ECM) in the lung^1–5^. Pulmonary fibrosis is a notably complex form of lung disease resulting from various factors^6^.

With the benefit of a wide range of targets, some traditional medications have been shown to be advantageous in the treatment of pulmonary fibrosis. Shen-mai-kai-fei-san (Shenks) is a Chinese herbal preparation that was developed by Yiling Hospital, affiliated to Hebei Medical University. Crucially, Shenks has been shown to be effective in the treatment of pulmonary fibrosis (including scleroderma-related fibrosis). However, mechanistic studies delineating the anti-fibrotic mechanisms behind Shenks treatment for pulmonary fibrosis remain scarce. Fibrogenesis is influenced by a variety of cytokines, among which transforming growth factor (TGF)-β is the most potent stimulator of collagen production^7^. Numerous studies have clarified the pathways of TGF-β involvement in the expression of extracellular matrix (ECM) genes as well as the pathogenesis of fibrosis^8–10^. Oxidant stress, which results from excessive ROS production and defects in, or the depletion of, antioxidant defenses, is one of the major mechanisms present in the pathogenesis of pulmonary fibrosis^11–14^. In cystic fibrosis patients, antioxidant defenses that are ordinarily capable of dealing with elevated oxidative stress are dysfunctional, leading to the occurrence of pulmonary cystic fibrosis^15^. Moreover, evidence has suggested that a causal agent of idiopathic pulmonary fibrosis (IPF) might be an imbalance between oxidant/antioxidant in the lungs of sufferers^13, 16, 17^. Finally, some antioxidant agents have the ability to prevent the development of experimental pulmonary fibrosis^18, 19^.

As it is compatible with the medicinal criteria termed “Jun, Chen, Zuo, Shi”, Shenks is composed of the following active ingredients: *Panaxquinquefolius*, *Ophiopogon japonicas*, *Salvia miltiorrhizaBge*, *Gynostemmapentaphyllum (Thunb.) Makino*, *AmygdalusCommunis Vas*, *Scutellari* + *abarbata D. Don, Lysimachiahui Diels*, and *Perillafrutescens*. Among these components in the Shenks formula, *Panaxquinquefolius*is the “Jun” medicine, or the principal component with the main therapeutic activity. *Ophiopogon japonicas*, *Salvia miltiorrhizaBge*, and *Gynostemmapentaphyllum (Thunb.) Makino* are the “Chen” medicines, or the secondary principal components of the formula used to enhance or assist the effect of the principal constituent. The rest are the “Zuo” and “Shi” components of the formula, and function to treat accompanying symptoms, enhance the delivery of herbal ingredients, and/or control the toxicity of the primary components^20, 21^. *Panaxquinquefolius*, with its anti-oxidative stress property, has also been reported to prevent glucose-induced injury in endothelial cells and H_2_O_2_-induced damage in rat lung cells^22, 23^. *Salvia miltiorrhiza*, a secondary principal component, was found to exert an anti-fibrotic effect and inhibit experimental skin fibrosis via a TGF-β signaling pathway^24^. In addition, its active component, salvianolic acid, is capable of attenuating liver fibrosis via TGF-β-related signaling pathways^25^. Thus, we speculate that the *Salvia miltiorrhiza* in the Shenks formula exerts its anti-fibrotic effect via TGF-β/Smad signaling. *Gynostemiapentaphyllum (Thunb.) Makino* has been reported to inhibit PDGF-induced type I procollagen expression^26^ and attenuate liver fibrosis^27^. Here, we propose that Shenks exerts an anti-fibrotic effect via both TGF-β/Smad signaling and anti-oxidant activities. Thus, the aim of the present study was to explore the mechanism of Shenks’ actions in the treatment of pulmonary fibrosis.

Both *in vivo* and *in vitro* studies were conducted to investigate the treatment effect of Shenks in pulmonary fibrosis, and found that Shenks inhibited fibrosis by blocking TGF-β pathway and balancing of oxidants and antioxidants.

## Results

### Shenks prevented pulmonary fibrosis in mice treated with bleomycin

The previous work has shown that pulmonary fibrosis can be successfully induced in mice using a single intratracheal instillation of bleomycin (BLM)^28^. It has been reported that after the administration of bleomycin, there is an onset of acute inflammation that can last up to 8 days that is followed by fibrogenic changes. With this timeline, treatments during the first seven days are considered preventive while treatments that occur during the later stages (>days 7–10) are considered therapeutic^29^. As reported by Tashkin, cyclophosphamide (CYC) is effective in the treatment of pulmonary fibrosis with scleroderma^30, 31^. In this study, CYC was selected as the positive control to evaluate the effects of Shenks.

**Figure 1:**
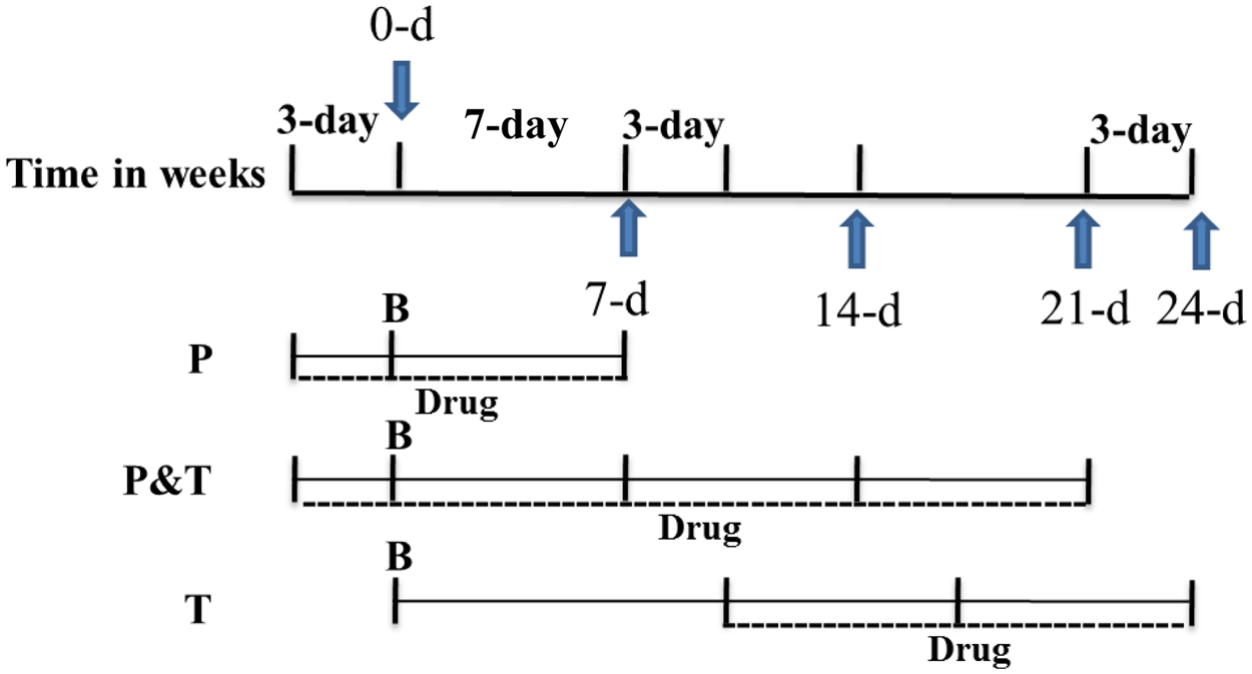
Schematic illustration experimental design. Mice were divided into three groups, bleomycin was instilled on Day 0 in all these three groups. Treatment drugs were administered 3 days before bleomycin instillation and till to Day 7 in P Group, were administered 3 days prior to bleomycin and up to Day 21 in P&T Group, and were given to the mice from Day 10 to 24 in T group. *P: Prevention*; *T: Therapy*; *B: Bleomycin*; *Drugs: Shenks or CYC*.

To determine the effect of Shenks on inflammation at the inflammation stage, Shenks and CYC were administered to BLM-treated mice three days before BLM and continued through to Day 7 (Prevention group, P group) as illustrated in Fig. 1. The results showed the number of the total inflammatory cells, neutrophils, lymphocytes, and macrophages, and the protein levels of the inflammatory cytokine IL-1β and IL-6 in BALF were remarkable elevated after bleomycin administered, but which were reduced by both Shenks and CYC treatment (Supplementary Fig. 1).

**Figure 2:**
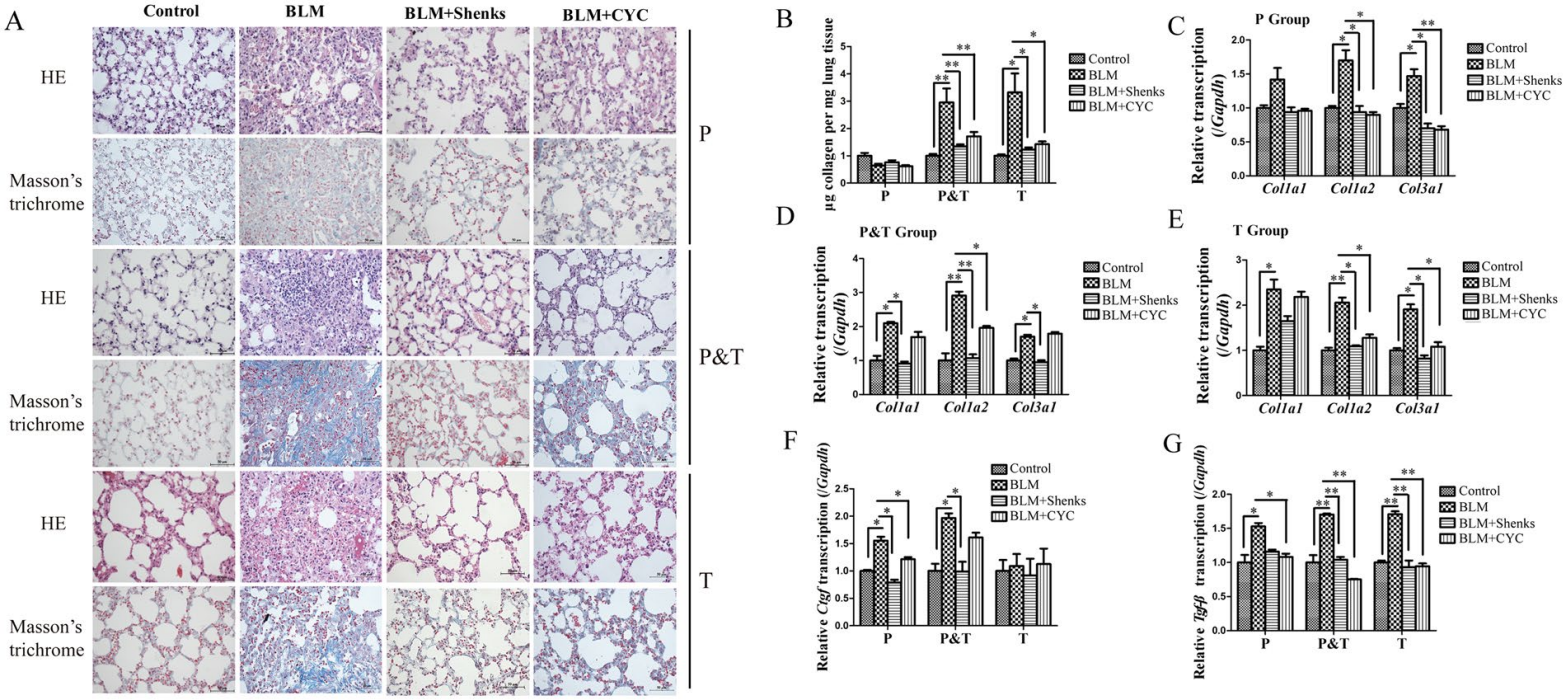
Shenks inhibition on bleomycin (BLM)-induced mice lung fibrosis. (**A**) Histological findings revealed by both H&E and Masson’s staining of lung inflammation and fibrosis in mice treated with saline and placebo, mice treated with bleomycin and placebo, and mice treated with bleomycin and Shenks or CYC. *Original magnification* x400. (**B**) Lung collagen content as determined by Sircol assay (n = 6 mice per group). (**C**,**D**,**E**) Collagen mRNA levels in murine lungs examined by RT-PCR. mRNA levels were calculated using a relative ratio to *Gapdh*. (**F**,**G**) *Ctgf* and *TGF-β* mRNA levels in murine lungs examined by RT-PCR. mRNA levels were calculated using a relative ratio to *Gapdh*. *Values in* (**B**,**C**,**D**,**E** and **F**) *are mean *± SEM from six mice per group. *P < *0.05*, **P < *0.001 when compared with mice treated with bleomycin and placebo. H&E* = *Hematoxylin and eosin*.

Examination on P and P&T (Prevention & Therapy group, BLM-treated mice three days before BLM through to 21 days after BLM) group were performed to examine the effect of Shenks on the development of pulmonary fibrosis when administered before fibrosis development. HE examination showed that, BLM-treated mice exhibited a significant disruption of the alveolar units and filtration of inflammatory cells into the interstitial and peribronchial of mice lungs in both P and P&T group, as well as remarked collagen deposited in the mice lung interstitial in P&T group as observed by Masson’s staining when compared with control mice (saline treatment alone). In contrast, after treatment with Shenks and CYC, the disruption of the alveoli was improved as indicated by decreased infiltration of inflammatory cells and reduced accumulation of ECM proteins, and the effect of Shenks was comparable with that of CYC (Fig. 2A, Supplementary Fig. 2). Compared with saline controls, BLM-treated mice had resulted in significant increase collagen content (up to 3.0- and 3.3-fold higher in the P and P&T groups, respectively). Collagen content was significantly reduced in the P and P&T groups, with application of both Shenks (54% and 63%, respectively) and CYC treatments (42% and 57%, respectively) (Fig. 2B). Furthermore, collagen transcription levels were examined using real-time PCR (RT-PCR). Compared to controls, BLM administration enhanced the transcription levels of *Col1a1*, *Col1a2*, and *Col3a1* (1.42 ± 0.17, 1.7 ± 0.14, and 1.47 ± 0.1 in P group, 2.1 ± 0.04, 2.91 ± 0.12, and 1.7 ± 0.06 in P&T Group, respectively). However, after treatment with Shenks, the increased levels were recovered nearly to the normal level in both the P and P&T groups (Fig. 2C,D). CYC treatment also resulted in a statistically significant reduction of collagen transcription but only in the P group (Fig. 2C,D).

The mRNA levels of some pro-fibrotic cytokines, such as CTGF and TGF-β were examined. Bleomycin treatment led to enhanced *Ctgf* mRNA transcription in both the P (1.55 ± 0.07, *P* < 0.05) and P&T (1.7 ± 0.08, *P* < 0.05), respectively. These increases were significantly decreased by Shenks treatment, to nearly normal levels, in both groups. Moreover, CYC treatment also led to reductions in *Ctgf* transcription in the P group (Fig. 2F, *P* < 0.05). Similarly, in response to bleomycin, there were increases in *Tgf-β* transcription increased in both the P (1.53 ± 0.05, *P* < 0.05) and P&T (1.70 ± 0.01, *P* < 0.001) groups. Enhanced *Tgf-β* mRNA expression was inhibited by both Shenks and CYC treatment (Fig. 2G).

Fibrosis began to develop on days 7–9 after the BLM challenge. To more accurately define the role of Shenks during the fibrotic stage, Shenks was administered daily from day 10 after the BLM challenge up until day 21 (Fig. 1, T group). Our results indicated that both Shenks and CYC treatment alleviated pulmonary fibrosis (Fig. 2A,B and E–G), as measured by histological alterations, collagen content, and ECM gene expression between control (BLM + saline) and treatments (BLM + Shenks and BLM + CYC) groups (Fig. 2). Collectively, these results demonstrate that Shenks can not only prevent fibrosis development, but also successfully attenuate established fibrosis.

### Shenks treatment in fibroblasts attenuated collagen production stimulated by exogenous TGF-β

**Figure 3:**
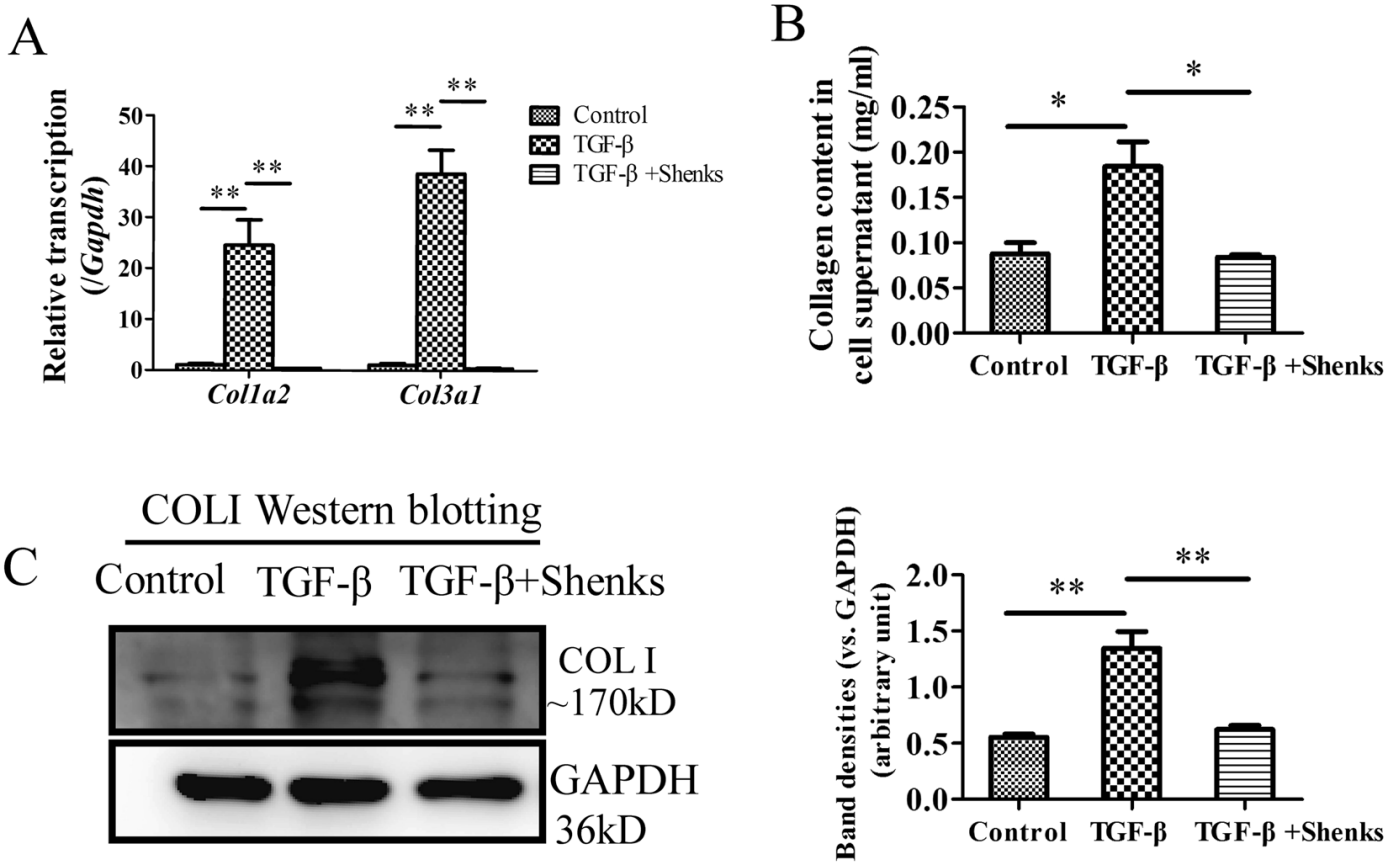
Shenks inhibition on collagen production. (**A**) Relative transcript levels of *Col1a2* and *Col3a1* in murine 3T3 fibroblasts that were exposed to different treatments. The expression level of each gene in the control group was normalized to 1. (**B**) Collagen content was determined by Sircol assay from the supernatants of cells that underwent different treatments. (**C**) Western blot analysis of type I collagen (COL1) in murine 3T3 fibroblasts that were exposed to different treatments. Densitometric analysis of Western blots for type I collagen (COL1) are shown. *Bars indicate the mean *± SEM results of three assays. *P < *0.05*; **P < *0.001. Control* = *PBS treated group*; *TGF-β* = *TGF-β-treated group*; *TGF-β* + *Shenks* = *TGF-β* ﻿*and﻿* *Shenks treated group*.

TGF-β stimulating NIH/3T3 fibroblasts were used to establish an *in vitro* model of pulmonary fibrosis in order to examine the anti-fibrotic efficacy of Shenks. As shown in Fig. 3, the *Col1a2* and *Col3a1* transcript levels were dramatically increased (24.6 ± 3.5, and 38.6 ± 3.3, respectively) after stimulation with exogenously applied TGF-β (Fig. 3A). However, Shenks treatment attenuated TGF-β-induced fibrosis and restored collagen gene expression to nearly normal levels (Fig. 3A). We then used a Sircol assay to measure the soluble collage protein content that had been secreted out of the cells. It indicated that exogenous TGF-β also induced increases in collagen secretion (52.4%, *P* = 0.006). This increase was then shown to be reduced by Shenks treatment to the normal level (51.8%, *P* = 0.007) (Fig. 3B). In addition, Western blot analysis showed that type I collagen production was highly activated by exogenous TGF-β (59.0%, *P* = 0.001) and subsequently decreased (53.8%, *P* = 0.001) after Shenks treatment (Fig. 3C). MRC-5, a human fetal lung fibroblast cell line, was also used to assess the effect of the Shenks on collagen production and TGF-β signaling. The results showed that the mRNA level of *Col1a2* and *Col3a1*, the soluble collage protein content determined by Sircol assay and the type I collagen production measured by western blotting were increased significantly by exogenous TGF-β treatment, whereas which were inhibited by Shenks treatment significantly (Supplementary Fig. 3). These data confirmed that Shenks is effective for fibrosis treatment.

### Shenks treatment attenuated type I collagen production via downregulation of Smad3 phosphorylation and SBE activity in type I collagen promoter

**Figure 4:**
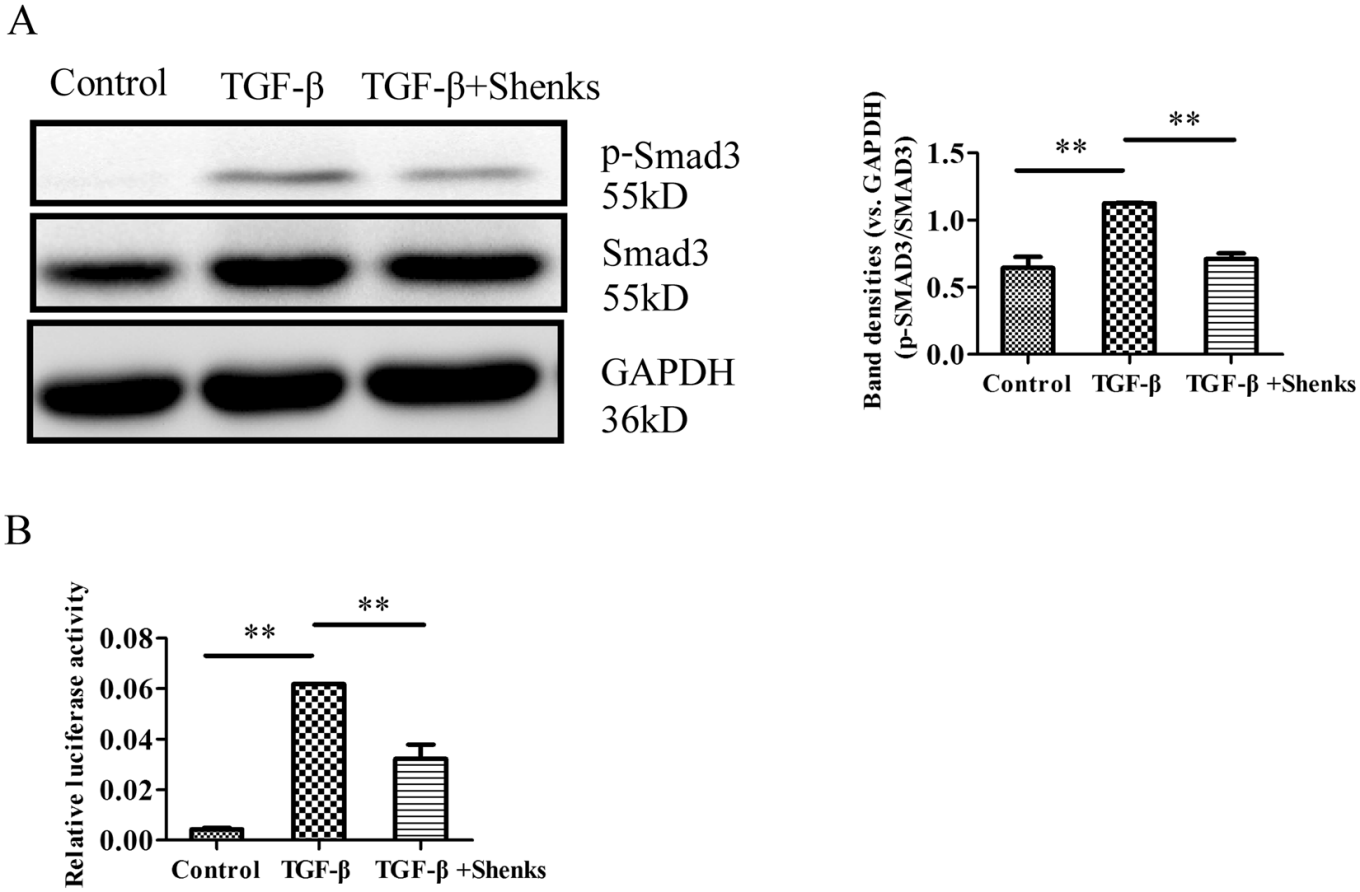
Shenks effect on pSmad3 and SBE. (**A**) Western blot analysis of pSmad3 and Smad3 protein content in murine 3T3 fibroblasts that underwent different treatments, and densitometric analysis of Western blots for p-Smad3 and Smad3. (**C**) Relative activity of SBE in the type I collagen promoter of murine 3T3 fibroblasts that underwent different treatments. The relative activity of SBE in non-treated murine 3T3 fibroblasts was normalized to 1. *Bars indicate mean *± SEM and are taken from experiments run in triplicate. *P < *0.05*; **P < *0.001*.

The TGF-β signaling pathway is the most potent, pro-fibrotic pathway characterized in the fibrogenesis of pulmonary fibrosis^11^. Smad proteins—particularly Smad3—are considered to be important signal transducers in TGF-β signaling. Furthermore, the phosphorylation of Smad proteins can result in signal transduction and transcriptional activation of target genes. To investigate whether Shenks had an effect on TGF-β signaling, we measured phosphorylation levels of Smad3 after the stimulation of exogenous TGF-β in different treatment groups. As shown in Fig. 4A, treatment with exogenous TGF-β led to a marked increase in p-Smad3 protein levels (1.75 ±  0.004-fold higher, *P* < 0.001). Subsequent Shenks treatment led to a lower phosphorylation level of Smad3 (40.0% lower, *P* < 0.001), indicating that Shenks treatment alleviated the abnormal activation of the TGF-β pathway in pulmonary fibrosis.

We next conducted a luciferase reporter gene assay to determine whether the effect of Shenks on the activity of Smad-binding element (SBE) sequence in the collagen promoter mediated the regulation of TGF-β signaling in collagen. As shown in Fig. 4B, SBE activity was significantly increased (14.4 ± 0.03-fold, *P* < 0.001) after stimulation with exogenous TGF-β. And Shenks treatment significantly attenuated this increase by 47.9% (*P* < 0.001). This further suggests that Shenks could effectively alleviate fibrosis by inhibiting the TGF-β pathway.

### Shenks inhibited oxidant stress in lung tissues from bleomycin-treated mice

Oxidant stress, especially reactive oxygen species (ROS), could promote the fibrogenic response, including the development of pulmonary fibrosis^32, 33^. In addition, it has been shown that ROS was primarily generated from mitochondria-derived ROS or NAD(P)H oxidase^34^. Ras-related C3 botulinum toxin substrate 1 (Rac1) plays a pivotal role in mitochondrial ROS production^34^. Additionally, a member of the NAD (P) H oxidase family, NOX4, could be stimulated by TGF-β to exacerbate the pulmonary fibrogenic response^35^. In contrast, genes such as extracellular superoxide dismutase (*Ec-sod*) which has enzymatic antioxidant properties and is capable of scavenging ROS, as well as glutamate-cysteine ligase catalytic subunit (*Gclc*) which is an enzyme involved in the process of *de novo* glutathione (GSH) synthesis and GSH play a critical role in lung antioxidant defense^36^. And GSSG is the oxidized glutathione, a product of GSH undergoing oxidation.

To demonstrate the anti-oxidative effect of Shenks, we used an intra- and extracellular sulfhydryl donor (N-acetyl-cysteine, NAC), which is capable to be against oxidant stress as an *in vivo* positive control. Previous work has shown that NAC displays antioxidant properties in the form of protection from experimentally induced pulmonary fibrosis^37^.

**Figure 5:**
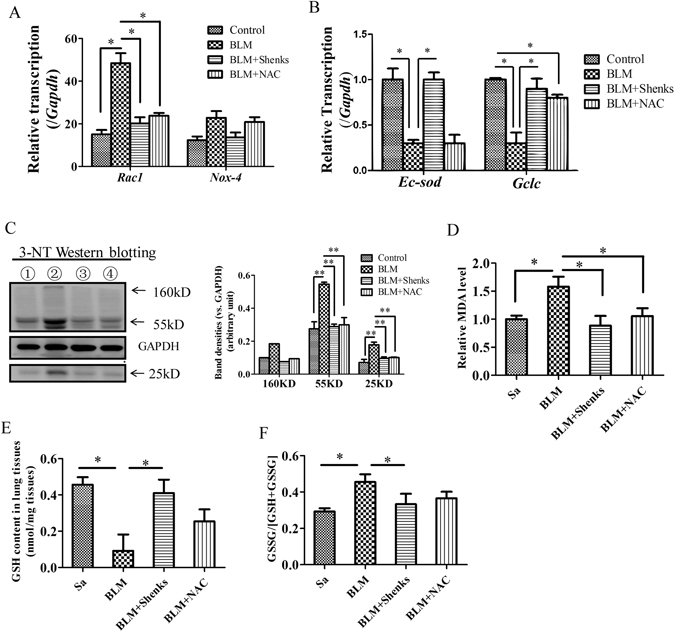
Modulation of Shenks on the balance of oxidant stress in the mice lungs. (**A**) Relative transcript levels of *Rac1* and *Nox-4* in lung tissues of mice that underwent different treatment conditions. The expression level of each gene in the control group was normalized to 1. (**B**) Relative transcription of *Ec-sod* and *Gclc* in the lungs of mice that underwent different treatments. (**C**) Western blot analysis of oxidative protein damage product 3-NT in the lungs of mice that underwent different treatments and Densitometric analysis of Western blots for 3-NT. (**D**) Lipid peroxidation was evaluated by measuring malondialdehyde (MDA). Changes in GSH concentration (**E**) and the ration of GSSG/(GSH + GSSH) among various groups (**F**). *In Figure* (*C*), *Line ①* = *Control*; *Line ②* = *BLM*; *line ③* = *BLM* + *Shenks*; *line ④* = *BLM* + *NAC. Bars indicate the mean* ± SEM results of three assays. *P < *0.05*; **P < *0.001. Control* = *Saline treated group*; *BLM* = *Bleomycin-treated group*; *BLM* + *Shenks* = *﻿﻿BLM and*﻿﻿﻿ *Shenks treated group* (*n* = *6*).

In our study, both Shenks and NAC significantly inhibited BLM-induced lung fibrosis (data not shown). As shown in Fig. 5, *Rac1* mRNA elevated about 2.8-fold (*P* < 0.05) in response to BLM stimulation, whereas Shenks inhibited *Rac1* transcription significantly (by 58.2%, *P* < 0.05), which was comparable with the inhibition of NAC (by 50.9%, Fig. 5A). After treatment with BLM, the transcription of *Nox4* increased about 1.9-fold, which was then could be inhibited by Shenks other than NAC, but no significant change was determined (Fig. 5A). In addition, we confirmed the level of NOX4 with immunohistochemical assay, and it was demonstrated that the NOX4 positive cell was slightly increased by bleomycin but which was reduced with Shenks treatment (Supplementary Fig. 4). Conversely, the anti-oxidative genes *Gclc* and *Ec-sod* expression levels decreased with application of bleomycin. And Shenks treatment led to a significant up-regulation in the expressions of these genes, which were comparative to levels seen with NAC treatment in the expression of *Gclc*, while NAC has little effect on the expression of *Ec-sod* (Fig. 5B).

Western blot analysis indicated that there was a marked up-regulation in the protein level of oxidative damage product 3-nitrotyrosine (3-NT) with bleomycin treatment (50%, *P* = 0.001), which is an important parameter of oxidative damage. Shenks treatment was found to significantly (*P* = 0.001) reduce the excessive production of 3-NT to near normal levels (Fig. 5C). To further investigate the anti-oxidative effect of Shenks, we measured the levels of malondialdehyde (MDA), a product of lipid peroxidation in mice lung tissues after Shenks treatment. As shown in Fig. 5D, both Shenks and NAC had significant inhibition effect on the overproduction of lipid peroxidation MDA induced by BLM (by 44.0% and 33.7%, respectively, *P* < 0.05), nearly to the normal level (*P* < 0.05). To elucidate whether bleomycin and Shenks treatment contribute to the concentration change of GSH, we determined the concentration of GSH and glutathione disulfide (GSSG) in mice lungs. The results showed that the concentration of GSH was decreased (lower 79.8%, *P* < 0.05, Fig. 5E), while the ratio of GSSG to GSSG + GSH was increased (about 1.55-fold, *P* < 0.05, Fig. 5F) by BLM challenge, however which returned to the normal by Shenks (Fig. 5E,F, *P* < 0.05), but the treatment effect of NAC had no significance (Fig. 5E). Taken together, these results suggests a pivotal role of oxidant damage in the development of pulmonary fibrosis. Importantly, Shenks is capable of regulating the balance of oxidant and anti-oxidant gene expressions to better protect against fibrosis.

### Shenks inhibited TGF-β-induced oxidative injury

**Figure 6:**
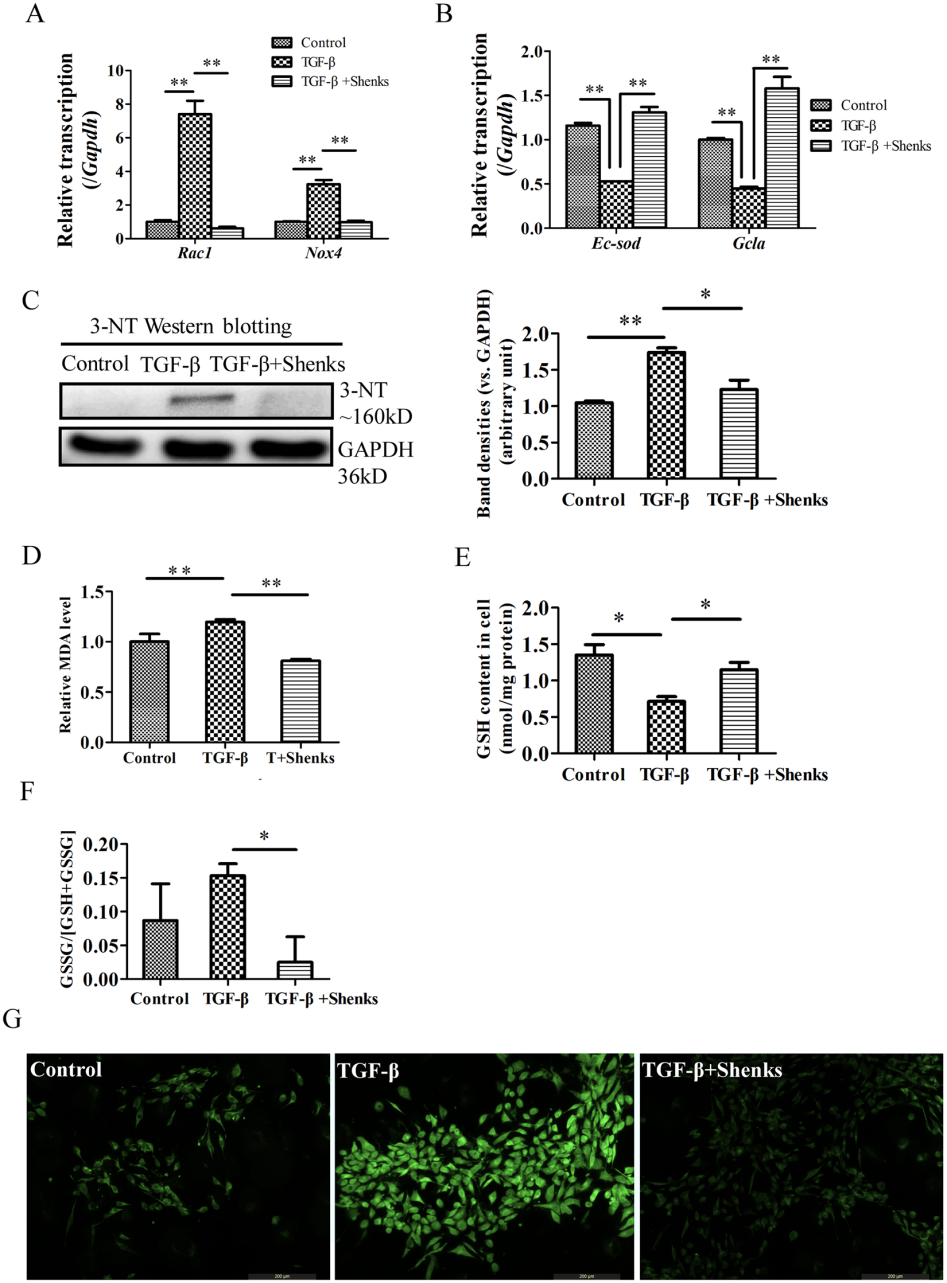
Shenks-mediated modulation of the balance of oxidant stress in TGF-β-stimulated cells. (**A**) Relative transcript levels of *Rac1* and *Nox-4* in murine 3T3 fibroblasts that underwent different treatment conditions. The expression level of each gene in the control group was normalized to 1. (**B**) The relative transcript levels of *Ec-sod* and *Gclc* in murine 3T3 fibroblasts which also underwent different treatments. The expression levels of each gene in the control group were normalized to 1. (**C**) Western blot analysis of 3-NT in differentially treated murine 3T3 fibroblasts and densitometric analysis of Western blot for 3-NT. **(D**) Lipid peroxidation MDA levels in murine 3T3 fibroblasts underwent different treatments. Changes in GSH concentration (**E**) and the ratio of GSSG/(GSH + GSSH) in murine 3T3 fibroblasts underwent different treatments (**F**). (**G**) Cells incubation with TGF-β and Shenks for 24 h was used to analyze the ROS status by using DCFH-DA as fluorogenic probe and visualization in a confocal microscope. *Bars indicate the mean* ± SEM results of three assays. *P < *0.05*; **P < *0.001*.

*In vitro* study was conducted to confirm the effect of Shenks on oxidant stress. TGF-β-challenged fibroblasts showed over-expression of oxidative genes, including *Rac1* and *Nox-4* (7.4 ± 0.8 and 3.2  ± 0.24, respectively, *P* < 0.001), while the anti-oxidant gene *Ec-sod* and *Gclc* was down-regulated (0.53 ± 0.00 and 0.45 ± 0.02, *P* < 0.001). In contrast, our results showed that Shenks treatment significantly inhibited both *Rac1* and *Nox-4* expression and increased *Ec-sod* and *Gclc* expression (Fig. 6A,B). Western blot analysis also indicated that 3-NT production was significantly activated upon exogenous application of TGF-β (39.9%, *P* = 0.001). This activation was reversed after Shenks treatment (29.3%, *P* = 0.005) (Fig. 6C). In addition, cellular MDA level was also increased remarkably by exogenous TGF-β stimulation (*P* < 0.001), and this increase was attenuated by Shenks (Fig. 6D). We also measured the levels of GSH and GSSG in fibroblasts stimulated by exogenous TGF-β with or without Shenks. Consistent with *in vivo* results, the concentration of GSH was decreased (lower 47.2%, *P* < 0.05, Fig. 6E), while the ratio of GSSG to GSSG + GSH was increased (about 1.1.77-fold, Fig. 6F) after TGF-β stimulation. Whereas, Shenks treatment enhanced the production of GSH nearly to the normal (*P* < 0.05, Fig. 6E) and inhibited the production of GSSG markedly (lower 83.70%, *P* < 0.05, Fig. 6F). To further assess the inhibition effect of Shenks on the production of ROS, ROS intracellular content was measured by using DCFH-DA as fluorescent probe. As shown in Fig. 6G, a representative image of the DCFH-DA fluorescence analysis revealed the increase in ROS content in TGF-β treated cells and decrease after Shenks administration. Taken together, these results further demonstrate that Shenks can regulate the balance of oxidant and antioxidant to prevent fibrosis.

## Discussion

The present study demonstrated that no matter whether Shenks was applied prophylactically or therapeutically, it was capable of protecting against bleomycin-induced pulmonary fibrosis in mice, which are comparable to those seen with CYC treatment. Furthermore, Shenks treatment significantly attenuated the collagen production in mice, and Shenks reduced the expression of TGF-β-stimulated collagen in cultured lung fibroblasts. Finally, applying Shenks led to an inhibition of fibrosis by modulation of TGF-β signaling and oxidant stress.

Normal lung tissues maintain an oxidant/antioxidant balance, which can eliminate the reactive oxygen species (ROS) that are inhaled or released by inflammatory cells. ROS is predominantly generated from either mitochondria-derived ROS and/or NAD (P) H oxidase. It was demonstrated Shenks could inhibit the expression of the oxidative genes *Nox4* and *Rac1* involved in the induction of ROS (Figs 5A and 6A), and further reduce the production of intracellular ROS induced by TGF-β (Fig. 6G). In fact, there exists an antioxidant system capable of scavenging ROS^38^, which includes enzymic antioxidants such as superoxide dismutase (SOD), catalase (CAT), and guaiacol peroxidase (GPX), as well as non-enzymatic scavengers such as glutathione (GSH), vitamin E, vitamin C, β-carotene, and flavonoids^39^. Extracellular superoxide dismutase (EC-sod) is localized to the extracellular matrix and the cell membrane and is one of the enzymic antioxidants. Previous work has shown that EC-sod is capable of preventing pulmonary fibrosis from bleomycin challenge^38, 40^. GSH can directly scavenge ROS through an oxidation/reduction reaction^39^. In the process of *de novo* GSH synthesis, glutamic cysteine ligase is the rate-limiting enzyme and is composed of glutamate-cysteine ligase catalytic subunit (GCLC) and glutamate-cysteine ligase modifier subunit (GCLM)^36^. It is also likely that increasing GCLC expression alone is able to induce GSH synthesis^36^. Our study revealed that Shenks can regulate oxidative stress balance by up-regulating the expression of anti-oxidative genes *EC-sod* and *Gclc* and down-regulating the expression of oxidative genes *Nox4* and *Rac1* (Figs 5A,B and 6A,B). As a result, Shenks reduced the oxidative production of protein 3-NT (Figs 5C and 6C) and lipid MDA (Figs 5D and 6D), as well as rescued the content of GSH (Figs 5E and 6E) in the BLM stimulated mice and TGF-β stimulated cell, and the anti-fibrotic ability was comparable with that of NAC (Fig. 5). Thus, Shenks’ ability to modulate oxidation balance might account for its activity to prevent fibrosis.

Fibrosis is the most severe manifestation of pulmonary fibrosis, which is caused by the over-production of collagen, and TGF-β is the acknowledged factor that has great pro-fibrotic potential by stimulating the expression of collagen. Over-expression of collagen was observed both in bleomycin-induced mice and TGF-β treated cells (Figs 2 and 3), while Shenks treatment in this study could modulate the collagen production to normal levels (Figs 2 and 3). We also found the inhibitory effect of Shenks on the activity of TGF-β signaling by reducing the phosphorylation levels of Smad3 and SBE activity (Fig. 4). On the other hand, TGF-β also mediates fibrogenesis through mediation of oxidant stress^41^. Increased TGF-β expression contributes to oxidant injury to promote the development of pulmonary fibrosis^36^. It was found that the expression level of the oxidative genes and 3-NT protein were increased by TGF-β in this study (Fig. 6C). Furthermore, TGF-β is capable of inhibiting the production of GSH and stimulating the production of MDA and reactive oxygen species (ROS) (Fig. 6D–G), which then potentiate TGF-β signaling and mediate fibrogenic responses stimulated by TGF-β^41, 42^. However, Shenks has the ability to modulate both TGF-β and oxidant stress whatever stimulated by bleomycin *in vivo* or stimulated by TGF-β *in vitro* (Figs 4 and 6).

In the situation of acute diseases, many modern drugs with good targetability and usually in the form of single-chemical entities are favorable due to their fast action and prediction. However, pulmonary fibrosis is a notably more progressive and irreversible form of lung disease. Such complex chronic conditions are significantly more difficult to be treated and usually serious side effects will occur due to the drug’s long term administration. Comparatively, Traditional Chinese Medicine (TCM), such as Shenks, is formulated from herb mixtures, in which several active ingredients are included in one prescription. These ingredients are aimed at numerous targets and, when combined, work either synergistically or antagonistically to yield a moderate effect. In this way, these traditional herbal “cocktails” can provide the best therapeutic benefit to the individuals^43^. In the present work, we found that Shenks can simultaneously inhibit the infiltration of inflammatory cells in murine lungs, decrease the collagen production and the expression of pro-fibrotic cytokines Tgf-β and Ctgf (Fig. 2, Supplementary Figs 1 and 2) and significantly reduce phosphorylation levels of Smad3 and SBE activity (Fig. 4), as well as modulate the oxidant/antioxidant balance in BLM-treated mice lungs and TGF-β stimulated cells (Figs 5 and 6). Since TCM emphasizes the maintenance and restoration of balance, it is much more suitable for the treatment of complex and chronic diseases. To this end, TCM has been shown to be effective in the treatment of some complex chronic disease, including type II diabetes^44^, promyelocytic leukemia^21^, and scleroderma^24^.

In summary, our results revealed that Shenks treatment for pulmonary fibrosis significantly reduced collagen production. This was shown both *in vivo* with a bleomycin-induced mouse model and *in vitro* using a TGF-β-induced NIH/3T3 fibroblast. This effect occurred via downregulation of Smad3 phosphorylation, downregulation of SBE activity, and modulation of the oxidant stress balance. This work indicates that Shenks had potential as a valid treatment approach for comprehensive conditions. By demonstrating that Shenks is an alternative treatment to conventional medicine for use in pulmonary fibrosis, our study provides valuable information for the development of new medications for fibrotic disorders.

## Methods

### Mouse model

A single intratracheal installation of bleomycin (2.5 U/kg) was used to induce lung fibrosis in C57BL/6 female mice (7 weeks old). Shenks (80 mg/d/mouse) and cyclophosphamide (CYC) (37.5 mg/kg) were dissolved in water and given daily to the mice, and the same volume saline was given to mice in control. Mice were divided into three groups: prevention (P), prevention plus therapy (P&T), and therapy (T). As shown in Fig. 1, in the prevention (P) group, drugs were administered daily to the mice three days before bleomycin stimulation through to 7 day after bleomycin to investigate the anti-inflammatory effect of Shenks; in the prevention & treatment (P&T) group, drugs were also administered daily to the mice three days before bleomycin and through to 21 day after bleomycin; and in the treatment (T) group, the drugs were given to the mice daily 10 days after bleomycin through to 14 day after bleomycin to determine the anti-fibrotic effect of Shenks. The present study was approved by and carried out in accordance with the guidelines of the School of Life Sciences, Fudan University.

### Histological analysis

To assess histopathological changes, lung tissue was fixed in 4% paraformaldehyde and embedded in paraffin wax. Tissue was sliced into 4-um-thick lung sections and then stained with either hematoxylin/eosin (H&E) or Masson’s trichrome staining for better visualization of the tissue structure. For the Immunohistochemical staining assay, the primary antibody used were anti-NOX4 (5 ug/mL) (Arigo biolaboratories, ARG55254). Mouse lung sections were deparaffinated and incubated with 5% bovine serum albumin for 60 minutes. Cells positive for NOX4 were detected by incubation with the primary antibody for 2 hours at room temperature followed by incubation with 3% hydrogen peroxide for 10 minutes. Goat anti-rabbit lgG labeled with horseradish peroxidase were used as secondary antibodies. The expression of NOX4 was visualized with 3,3′-diaminobenzidinetetrahydrochloride (DAB-4HCl). Changes in pulmonary tissue were analyzed using a Nikon Eclipse 80i microscope (Nikon, Badhoevedorp, the Netherlands). Tissue evaluation was performed by two independent examiners.

### Collagen measurements

Total soluble collagen from mouse lungs was quantified using a Sircol collagen assay and according to the manufacturer’s instructions (Biocolor, Belfast, UK). In brief, collagen was extracted and digested overnight with 2 mg/ml pepsin in 5 M acetic acid. Digested collagen solution (20 μl) was then added in Sirius red dye (1 mL), which is an anionic dye that reacts specifically with the basic side chain groups found in collagen. The resulting solution was incubated at room temperature for 30 min and under gentle rotation. After centrifugation at 12,000  for 10 min, the collagen-bound dye was re-dissolved with 1 ml of 0.5 M NaOH. The absorbance at 555 nm was then measured, as it is directly proportional to the amount of collagen present in lung tissue.

### Cell culture and exposure to TGF-β

Mouse embryonic NIH/3T3 and human fetal lung MRC-5 fibroblasts were cultured at 37 °C in a 5% CO_2_ humidified environment and in DMEM supplemented with 10% fetal calf serum. Fibroblasts were then placed into 12-well culture plates at a density of 1 × 10^5^ cells per well for later gene and protein expression assays. After incubating for 12 h in a serum-free media to induce serum starvation, fibroblasts were exposed to recombinant TGF-β (R&D Systems, Inc., Minneapolis, MN, USA) at a concentration of 10.0 ng/ml for 24 h (RNA collection) and 48 h (protein collection).

### Luciferase reporter gene assay

pGL3-SBE4-Luc was a generous gift from Dr. Kiyoshi Higashi (Sumitomo Chemical Co., Ltd., Osaka, Japan) and consists of four short tandem repeats of the Smad binding element (SBE) (GTCTAGAC) and a minimal promoter (TATA box). The pRL-SV40 plasmid was used as an internal control. NIH/3T3 fibroblasts were placed into a 24-well culture plate and 500 ng of either pGL3-SBE4-Luc or pGL3-Basic were co-transfected with 10 ng of pRL-SV40 using Lipofectamin 2000 (Invitrogen, Carlsbad, CA, USA) and according to the manufacturer’s protocol. After 6 h post-transfection, culture medium was replaced by TGF-β medium that was either with or without Shenks. Cell lysates were harvested 24 h later and the luciferase activity assay performed according to the manufacturer’s instructions (Dual-luciferase Reporter Assay System, Promega, Madison, WI, USA). A GloMax 20/20 Luminometer (Promega, Madison, WI, USA) was used to assay luciferase output.

### Phosphorylation assay

Antibodies to Smad3 and p-Smad3 were purchased from Cell Signaling Technology Inc. (Beverly, MA, USA). NIH/3T3 fibroblasts were placed into a 12-well culture plate at a density of 1 × 10^5^ cells per well. Shenks was added to the culture medium and fibroblasts were cultured for 24 h. NIH/3T3 fibroblasts were then treated with TGF-β for 30 min, after which cell lysates were harvested for Western blot analysis.

### Real-time quantitative PCR (RT-PCR) analysis

Total RNA was extracted from murine lung and fibroblasts usingTrizol (Invitrogen, Carlsbad, CA, USA). Total RNA (1 μg) was subjected to cDNA synthesis using the High Capacity cDNA Reverse Transcription Kit (Applied Biosystems, Foster City, CA, USA) and according to the manufacturer’s instructions. The specific primers for each gene were designed using Primer 5 and synthesized by Generay Biotech Co., Ltd. (Shanghai, China). RT-PCR amplification was conducted using a SYBR Green I PCR Kit (TaKaRa, Shiga, Japan) and according to the manufacturer’s instructions. The reaction was carried out using an ABI Prism 7900 Detector System (Applied Biosystems). RT-PCR thermocycling conditions were as follows: 95 °C for 3 min, 40 cycles of 95 °C for 15 s, and 60 °C for 40 s. To generate the the dissociation curve, the parameters used were as follows: 95 °C for 15 s, 60 °C for 15 s, and 95 °C for 15 s. All resulting data were analyzed using SDS 2.3 software (Applied Biosystems). For each sample, the relative gene expression was calculated using a ratio of a known housekeeping gene, *Gapdh/GAPDH*.

### Western blot analysis

Cell lysates extracted from murine lung tissue and cultured cells were used for subsequent immunoblot analyses. Total protein concentration was measured using a BCA protein kit (Vazyme, China). Equal amounts of protein from each sample were subjected to 10% SDS PAGE gels electrophoresis after which protein was transferred to PVDF membranes (Millipore, Billerica, MA, USA). Membranes were blocked in 5% milk in TBST at room temperature for 1 h after which they were incubated with one of the following primary antibodies: mouse monoclonal [2A12] to 3-Nitrotyrosine (1:1000) (Abcam, Hong Kong, Ltd.), rabbit anti-mouse anti-Collagen type I polyclonal (1:500) (Millipore), or internal control GAPDH (1:5000–10000) (Vazyme, China) at 4 °C overnight. Membranes were washed three times with TBST for a total of 30 minutes and then incubated with the horse-radish peroxidase-conjugated secondary antibody of goat anti-rabbit, rabbit anti-goat, or goat anti-mouse lgG for 1 h at room temperature. The protein bands were visualized with ECL solution.

### Measurement of MDA

The lipid peroxidation product of malondialdehyde (MDA) generated in lung by free radical injury was measured by thiobarbituric acid (TBA) reactivity using the commercial colorimetric assay kit (Sigma-aldrich, Louis, USA). The concentration of MDA was calculated by a calibration curve using MDA.

### Measurement of GSH and GSSG Content

Total glutathione (GSH) and oxidative glutathione (GSSG) levels were measured by the colorimetric microplate assay kits (Beyotime, Nanjing, China). Briefly, lung tissues homogenates was centrifuged at 10,000 g for 10 min at 4 C. The supernatant was used for GSH and GSSG assay. The total GSH level was measured by the method of DTNB-GSSG recycling assay^45^. The GSSG level was quantified by the same method of total GSH assay after the supernatant was pretreated with 1% 1 mol/L 2-vinylpyridine solution to remove the reduced GSH. The amount of reduced GSH was obtained by subtracting the amount of GSSG from that of the total GSH.

### Measurement of intracellular ROS

The intracellular ROS was measured with 2′-,7′-dichloro-fluorescin diacetate (DCFH-DA; Sigma-Aldrich). After incubation of cells in the absence or presence of the different factors for 24 h, they were washed twice with phosphate-buffered saline (PBS), and then incubated with 10 μM DCFH-DA at a 37 °C humidified incubator for 30 min and washed twice with PBS. The cellular fluorescence intensity was visualized with fluorescence microscope (Olympus, Jap) after 30 min of incubation with 5 μmol/l DCFH-DA.

### Statistical analysis

Data are expression as mean ± SEM. Either an independent two group t-test or one-way ANOVA test with *post hoc* LSD’s multiple comparison test were used for the evaluation of significance between different groups. A *P* value of less than 0.05 was considered statistically significant.

## Electronic supplementary material

Supplemental Dataset
