# Supplementary material for: Treatment effects of the traditional Chinese medicine Shenks in bleomycin-induced lung fibrosis through regulation of TGF-beta/Smad3 signaling and oxidative stress
Source: Sci Rep. 2017 May 22;7:2252. doi: 10.1038/s41598-017-02293-z (PMC5440393; doi:10.1038/s41598-017-02293-z)
Supplement: Supplementary file 1 — Supplemental Dataset [file 41598_2017_2293_MOESM1_ESM.doc]

**Treatment effects of the traditional Chinese medicine Shenks in bleomycin-induced lung fibrosis through regulation of TGF-β/Smad3 signaling and oxidative stress**

Haiyan Chu^1#^, Ying Shi^2,3#^, Shuai Jiang^1#^, Yongqiang Zhao^2,3^, Qicheng Zhong^2^, Qingmei Liu^1^, Yanyun Ma^1^, Xiangguang Shi^1^, Weifeng Ding^1^, Xiaodong Zhou^4^, Jimin Cui^2^, Li Jin^1^, Gang Guo^2*^, Jiucun Wang^1,5*^

^1^State Key Laboratory of Genetic Engineering, Collaborative Innovation Center for Genetics and Development, School of Life Sciences, Fudan University, 2005 Songhu Road, Shanghai 200438, P.R.China;

^2^Department of Rheumatology and Immunology, Yiling Affiliated Hospital of Hebei Medical University, Shijiazhuang 050091, China;

^3^Department of Traditional Chinese Medicine, Geriatric Hospital of Hebei Province, Shijiazhuang 050011, China;

^4^University of Texas Health Science Center at Houston, 6431 Fannin St. Houston, Texas 77030, USA;

^5^Institute of Rheumatology, Immunology and Allergy, Fudan University, Shanghai 200040, P. R. China;

#: These authors contributed equally

*: Correspondence to: Dr. Jiucun Wang, or Dr. Gang Guo

Email addresses:

HC: [chuhaiyan1987@163.com](mailto:chuhaiyan1987@163.com)

YS: shiying070618@gmail.com

SJ: [zhiyusang@163.com](mailto:zhiyusang@163.com)

QZ: [zhqich90@163.com](mailto:zhqich90@163.com)

YZ: [zhaoyongqiangts@163.com](mailto:zhaoyongqiangts@163.com)

QL: [liuqing.mei@163.com](mailto:liuqing.mei@163.com)

YM: mayymail@163.com

XS: 14110700088@fudan.edu.cn

WD: dingweifeng.ntu@163.com

XZ: [Xiaodong.zhou@uth.tmc.edu](mailto:Xiaodong.zhou@uth.tmc.edu)

JC: 1245158450@qq.com

LJ: [lijin@fudan.edu.cn](mailto:lijin@fudan.edu.cn)

GG:chenxiangmahuang@163.com

JW: [jcwang@fudan.edu.cn](mailto:jcwang@fudan.edu.cn)

Running title: Treatment effect of Shenks on pulmonary fibrosis

**Supplementary Figure 1**


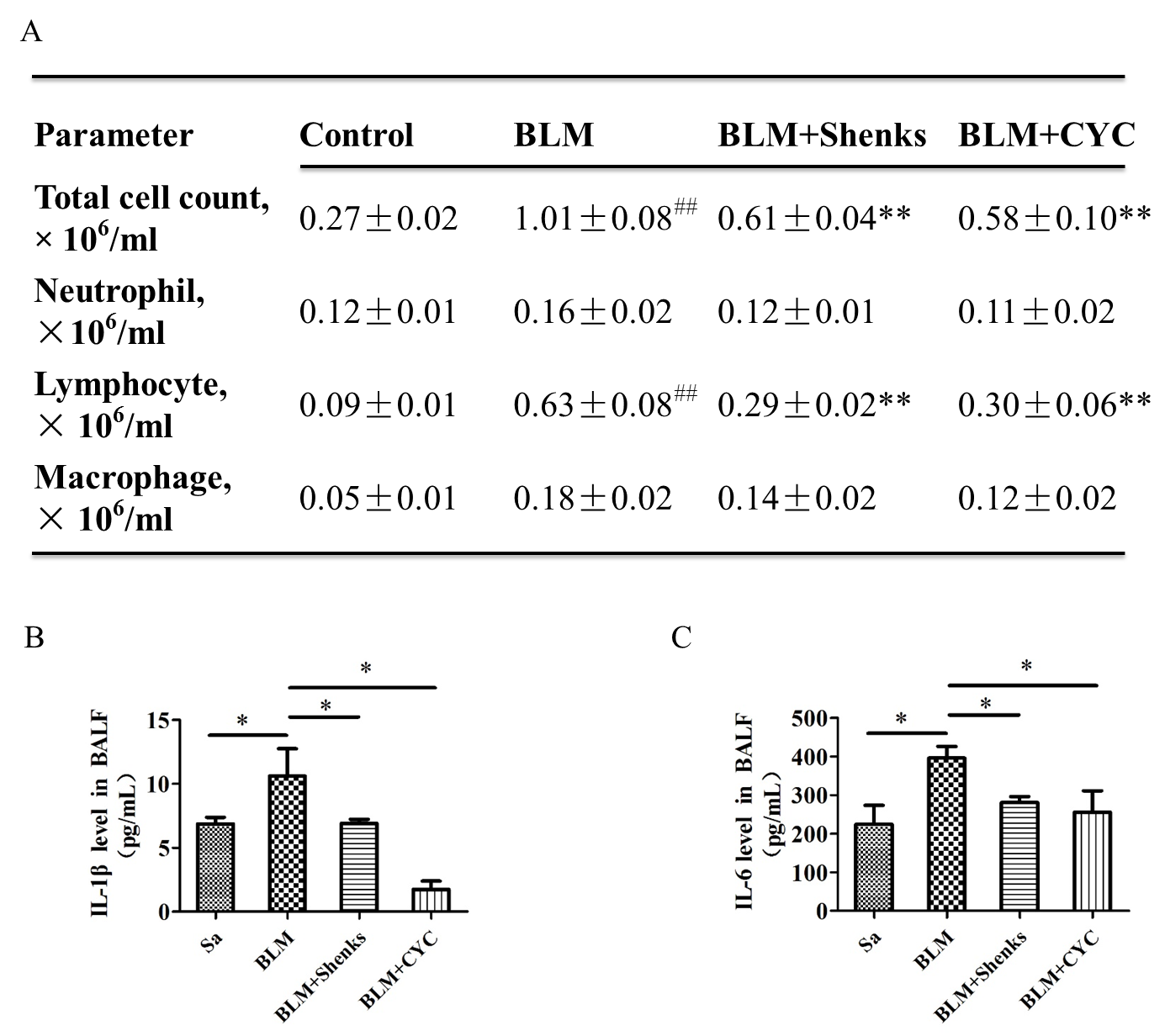
**Supplementary Figure 1. Shenks attenuates inflammation induced by bleomycin in mouse lung tissues.** (A) Cells number were counted in the BALF of mice. IL-1β (B) and IL-6 (C) BALF levels were determined by ELISA. *##P<0.001 versus mice treated with saline plus vehicle and **P<0.001versus mice treated with bleomycin plus vehicle in (A). Values are expressed as mean ± SEM, *P<0.05 versus mice treated with bleomycin plus vehicle in (B) and (C).*

**Supplementary Figure 2**

**
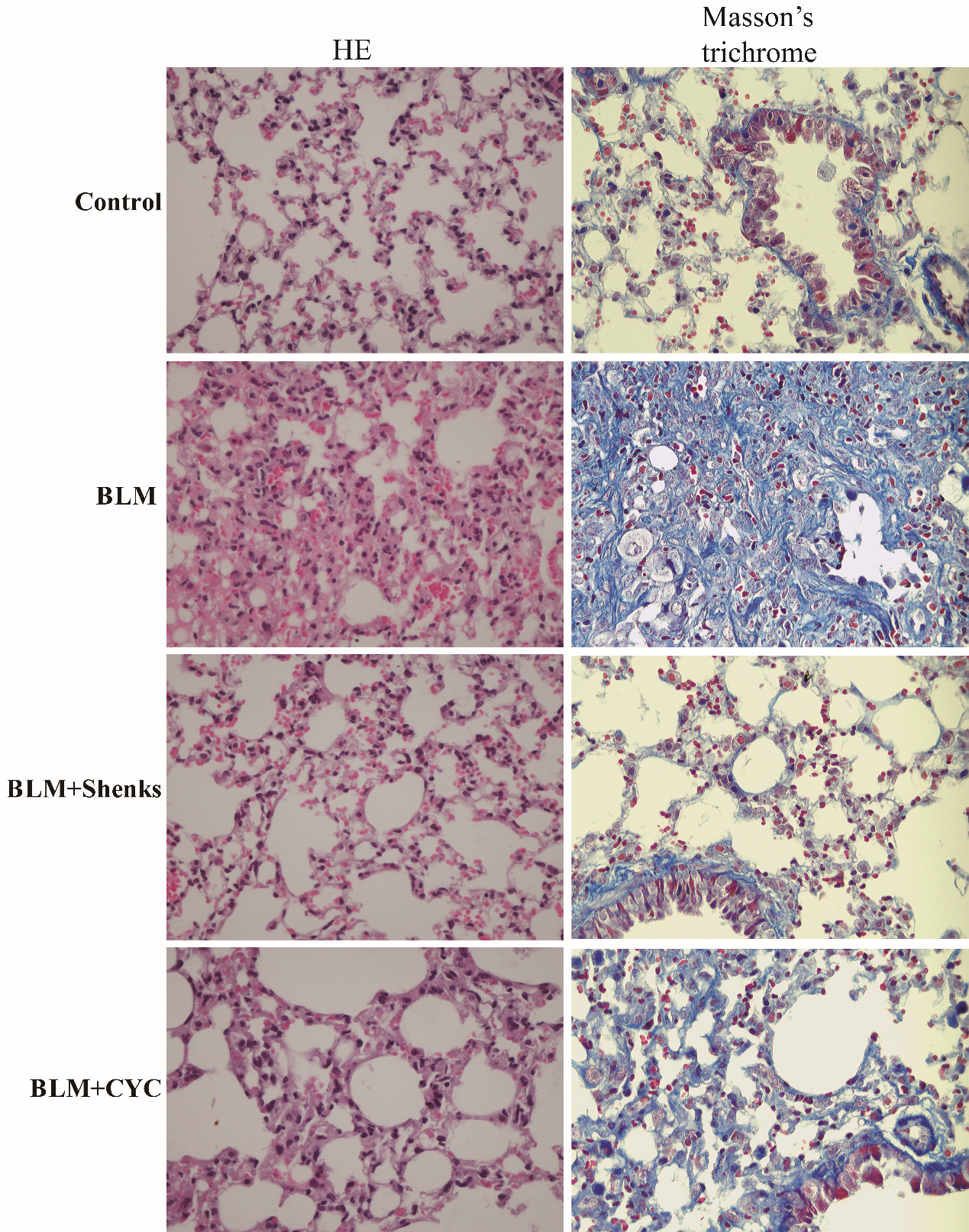
**

**Supplementary Figure 2. Shenks inhibition on bleomycin (BLM)-induced mice lung fibrosis**. Histological findings revealed by both H&E and Masson’s staining of lung inflammation and fibrosis in mice treated with saline and placebo, mice treated with bleomycin and placebo, and mice treated with bleomycin and Shenks or CYC. *Original magnification × 400*.

**Supplementary Figure 3**


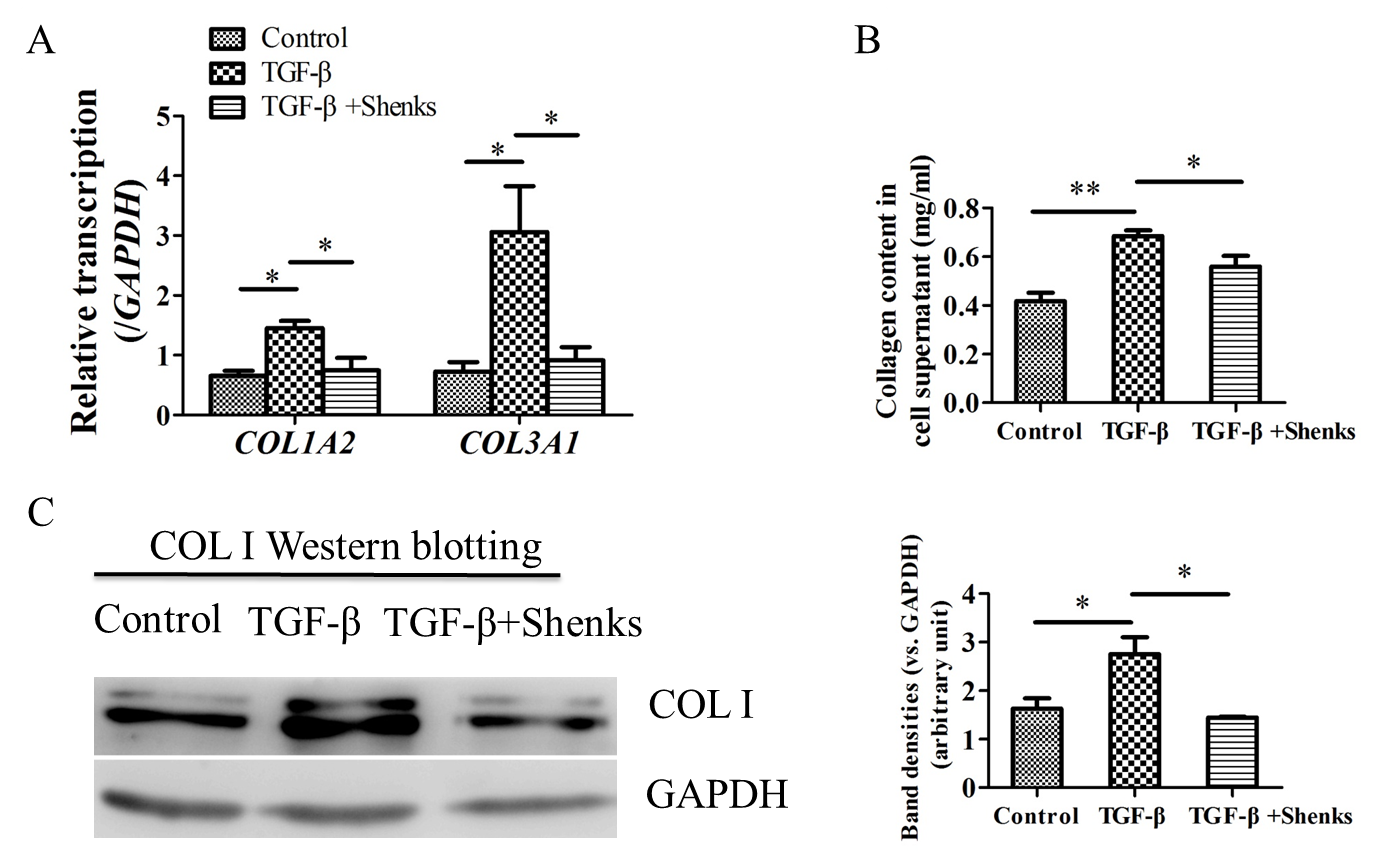


**Supplementary Figure 3. Shenks inhibition on collagen production in MRC-5 fibroblasts.** (A) Relative transcript levels of COL1A2 and COL3A1 in MRC-5 fibroblasts that were exposed to different treatments. The expression level of each gene in the control group was normalized to 1. (B) Collagen content was determined by Sircol assay from the supernatants of cells that underwent different treatments. (C) Western blot analysis of type I collagen (COL1) in MRC-5 fibroblasts that were exposed to different treatments. Densitometric analysis of Western blots for type I collagen (COL I) are shown. *Bars indicate the mean ± SEM results of three assays***.** **, P<0.05; **, P<0.001.*

**Supplementary Figure 4**

**
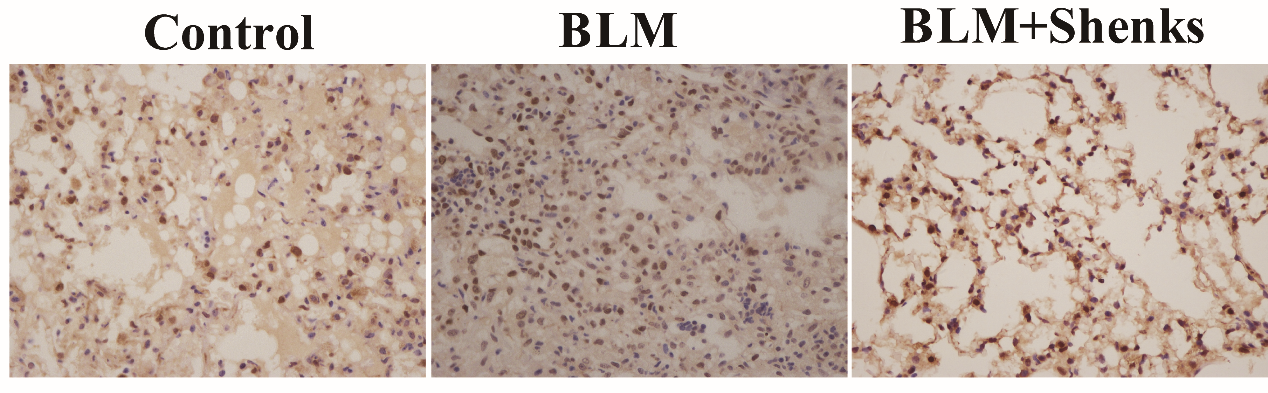
**

**Supplementary Figure 4. Immunohistochemical staining for NOX4 in mouse lung**. Mouse lung section from different treatment group was staining for NOX4 protein. The positive cell was observed with brown color. *Original magnification × 400*.
